# SOCIAL DETERMINANTS OF HEALTH RELATED TO GENREAL PHYSICIAN VISITS IN OLDER ADULTS LIVING WITH MULTIMORBIDITY

**DOI:** 10.1093/geroni/igad104.3088

**Published:** 2023-12-21

**Authors:** Chitchanok Benjasirisan, Arum Lim, Xiaoyue Liu, Oluwabunmi Ogungbe, Cheryl Dennison Himmelfarb, Patricia Davidson, Binu Koirala

**Affiliations:** Johns Hopkins University, Baltimore, Maryland, United States; Johns Hopkins University, Baltimore, Maryland, United States; Johns Hopkins University, Baltimore, Maryland, United States; Johns Hopkins University, Baltimore, Maryland, United States; Johns Hopkins University, Baltimore, Maryland, United States; University of Wollongong, Wollongong, New South Wales, Australia; Johns Hopkins University School of Nursing, Baltimore, Maryland, United States

## Abstract

Multimorbidity- the presence of two or more chronic conditions- is a global concern and is commonly associated with aging. Multimorbidity results in diminished physical function, higher healthcare utilization, and increased mortality rates. To understand healthcare challenges and increase prevention and continuity of care among older adults living with multimorbidity, the social determinants of health (SDoH) affecting general physician visits need to be explored. This study aimed to examine SDoH associated with general physician visits among 50 years and older adults living with multimorbidity using cross-sectional data from the 2010 to 2018 National Health Interview Survey. The SDoH, including marital status, race/ethnicity, education, poverty-income ratio, employment, health insurance, and having a usual place for medical care, were explored using logistic regression. The outcome was at least one visit to the general doctor’s office, a clinic, or elsewhere in the last 12 months. Among 25,108 participants (mean age ± SD was 68.1±10.16 years, 55.5% female, with 2.7±0.95 average number of self-reported morbidities) adjusted analysis controlling for age, sex, and categories of comorbidities found that Hispanic (OR=0.78, 95%CI=0.66-0.91), living below the poverty level (OR=0.79, 95%CI=0.68-0.92), and uninsured (OR=0.52, 95%CI=0.42-0.63) had lower odds of having one or more physician visits. Being unemployed (OR=1.26, 95%CI=1.11-1.42) and having a usual place for medical care (OR=7.94, 95%CI=6.39-9.86) had higher odds of having one or more physician visits. The result suggested that SDoH should be considered to navigate targeted health programs and policies to improve prevention and management of multimorbidity as well as to promote health equity.

